# Propagation of pure fetal and maternal mesenchymal stromal cells from terminal chorionic villi of human term placenta

**DOI:** 10.1038/srep10054

**Published:** 2015-05-15

**Authors:** Smitha Mathews, K. Lakshmi Rao, K. Suma Prasad, M. K. Kanakavalli, A. Govardhana Reddy, T. Avinash Raj, Kumarasamy Thangaraj, Gopal Pande

**Affiliations:** 1 CSIR-Centre for Cellular and Molecular Biology, Uppal Road, Hyderabad 500007, India; 2 Sridevi Nursing Home, Warasiguda, Hyderabad 500361, India; 3 Prasad Hospital and Research Centre, Nacharam, Hyderabad 500076, India

## Abstract

Long term propagation of human fetal mesenchymal stromal cells (MSC) *in vitro* has proven elusive due to limited availability of fetal tissue sources and lack of appropriate methodologies. Here, we have demonstrated the presence of fetal and maternal cells within the tips of terminal chorionic villi (TCV) of normal human term placenta, and we have exploited inherent differences in the adhesive and migratory properties of maternal *vs*. fetal cells, to establish pure MSC cultures of both cell types. The origin and purity of each culture was confirmed by X-Y chromosome-specific fluorescence *in situ* hybridization (FISH) and short tandem repeat (STR) genotyping. This is the first demonstration of fetal and maternal cells in the TCV of human term placenta and also of deriving pure fetal MSC cultures from them. The concomitant availability of pure cultures of adult and fetal MSC from one tissue provides a good system to compare genetic and epigenetic differences between adult and fetal MSCs; and also to generate new models of cell based therapies in regenerative medicine.

Placenta is a feto-maternal organ essential for the normal growth and development of the fetus^1^. Being hemochorionic in origin, the human placenta exhibits a more complex organization than others; at full term it has two well demarcated sides, maternal and fetal, which are internally separated by the chorionic and basal plates. The external appearances of maternal and fetal sides are very different. A branched network of fetal blood vessels and a network of relatively thicker chorionic villi generated exclusively by fetal trophoblasts are observed on the fetal side of the placenta, whereas, on the maternal side, thin endings of TCV are seen that are enshrouded by a highly vascularized decidua containing maternal cells (Supplementary Fig.S1).

Mesenchymal stromal cells (MSC) have been demonstrated and cultured from placental tissue isolated at early pregnancy (around 12 days post fertilization) and up to the full term placenta^2^. Placental MSC (PMSC) reside in a perivascular niche throughout pregnancy^3^ and play a significant role in the structural and functional development of the placenta particularly during vasculogenesis, angiogenesis and the villus branching^2^. It is interesting to note that so far all PMSC cultures from term placenta have been of maternal origin^4^ although fetal cells were demonstrated in some cultures in the early stages but they did not survive^5^,^6^.

There has been a growing interest in isolating fetal MSC because it is assumed that they would exhibit better *in vitro* proliferation and differentiation capacity when compared with adult MSC, due to their younger age^7^,^8^. Attempts to establish pure and stable fetal MSC cultures from placental tissues have not succeeded so far^9^,^10^ however, derivation of fetal MSC cultures from non-placental birth associated tissues such as amniotic membranes, amniotic fluid and Wharton’s jelly have been successful^11^. These tissues however are either limited in quantity or difficult to access. This report shows that TCV of term placenta could be an abundant and easily accessible source for obtaining fetal MSC.

Based on reports, so far^12^, TCV are known to contain only fetal cells whereas maternal cells are present in the inter-villus spaces. Here we have demonstrated, for the first time, the presence of maternal cells along with fetal cells within the same TCV and we have established pure fetal and maternal MSC cultures from that tissue. In order to establish the purity and cell origin of the cultures we used TCV of male babies, and employed short tandem repeat (STR)-based genotyping and X-Y chromosome specific fluorescence *in situ* hybridization (FISH) analysis. Both fetal and maternal cultures exhibited all the properties of MSC prescribed by the International Society of Cell Therapy Guidelines and further analysis of their molecular properties could be useful in understanding the plasticity of MSC derived from adult vs. fetal cells.

## Results

### Cell types in TCV

**Figure 1:**
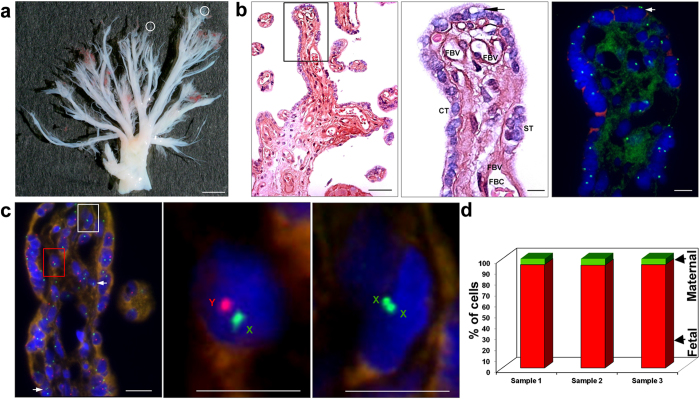
Gross anatomy and cellular features of terminal chorionic villi (TCV) isolated from a human placenta at full term. (**a**) Chorionic villi after the removal of maternal decidua (Scale bar, 3 mm). Maternal and fetal cells were isolated from small pieces from the encircled termini (1 mm^2^ area). (**b**) H&E stained histological features in a longitudinal section of the terminal villi and their matched male/female cell specific FISH analysis (a similar analysis of cells in the transverse section is shown in Supplementary Figure S2). Left panel shows the lower magnification (scale bar, 50 μm) and the right panels show the boxed region at higher magnification (scale bar, 10 μm). Cytotrophoblast (CT), syncytiotrophoblast (ST), fetal blood vessel (FBV), fetal blood cells (FBC) and a maternal cell within the cytotrophoblast layer are seen. (**c**) High resolution FISH-based detection of male fetal and maternal cells in a longitudinal section of a terminal chorionic villus (a similar analysis in the transverse section of the villi is shown in Supplementary Figure S2); Left panel shows the lower magnification (scale bar, 50 μm) and the right panels show the boxed region at higher magnification (scale bar, 10 μm). Arrows show two more maternal cells in the villus. (**d**) The percentage distribution of maternal (green, 5%) and male fetal cells (red, 95%) obtained from the X and Y specific-chromosome FISH analysis (n = 3).

Term placentas of male (n = 3) or female (n = 3) babies were collected and TCV from the maternal sides of the placenta, that had been cleared totally of the maternal decidua, were isolated for further analysis (Fig. 1a). H&E staining revealed the presence of cytotrophoblasts, syncytiotrophoblasts, fetal blood vessels and fetal blood cells within the TCV (Fig. 1b). A matched serial section of the same TCV region, processed for X and Y chromosome-specific FISH analysis, clearly showed the presence of maternal (XX) cells in the male (XY) TCV (Fig. 1c). High-resolution FISH analysis revealed the presence of maternal cells within the cytotrophoblast layer and near the fetal blood vessel of the TCV (Fig. 1c). Majority of the cells in the TCV were of fetal origin. A count of maternal *vs*. fetal cells in the TCV from 3 different male placentas showed that about 3-5% cells were of maternal origin (Fig. 1d).

**Figure 2:**
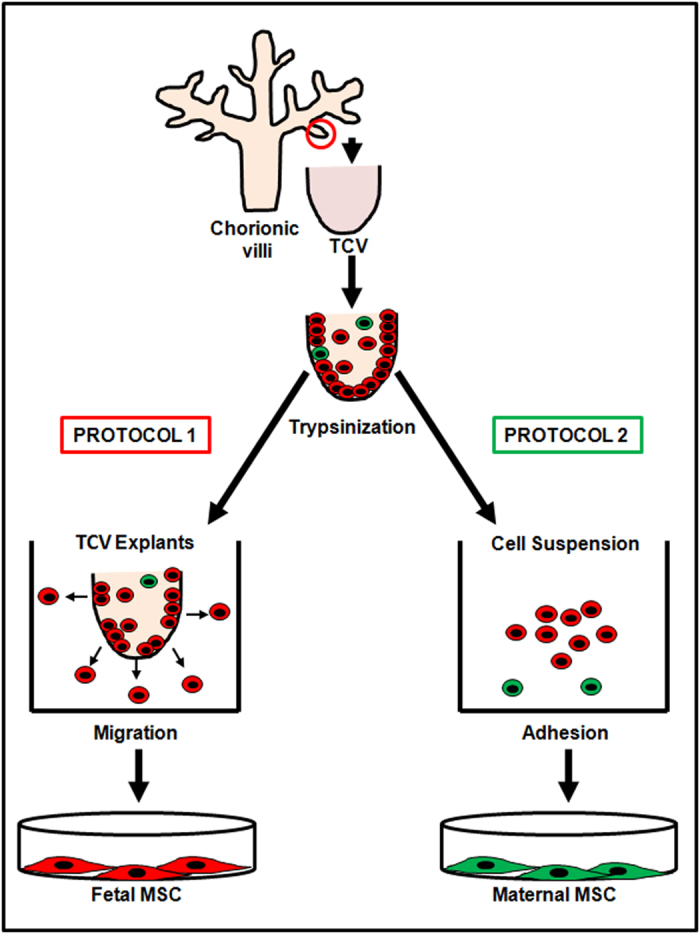
Schematic representation of 2 protocols for differential culture of MSCs of maternal and fetal origin. Protocol 1 was used for isolating fetal MSC and Protocol 2 for maternal MSC.

Two different protocols were used for isolating pure cultures of fetal and maternal stem cells (Fig. 2). Explants culture, according to protocol 1, generated fetal origin MSC; whereas selective adhesion of MSC by protocol 2 resulted in maternal origin MSCs.

### Fetal MSC characterization

**Figure 3:**
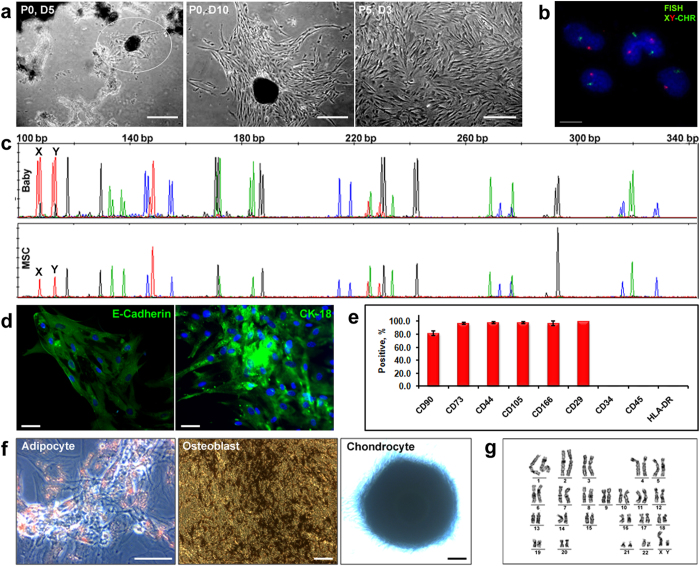
Cellular and molecular properties of MSC cultures established by Protocol 1. (**a**) Phase contrast images, on 5 days (P0, D5) and 10 days (P0, D10) after placing the explants. TCV attained spheroid like morphology in culture and the cells are seen migrating out of this spheroid (encircled area) (Supplementary online video S1 and S2). Fetal MSC cultures at passage 5 (P5, D3) are also shown (scale bars, 100 μm). (**b**) FISH images of migratory cell cultures at day 3 of passage 1 (scale bar, 10 μm). A similar FISH analysis was also done at passage 5 (Supplementary Figure S3). (**c**) Electropherograms showing the perfectly matching STR profile of the MSC (Passage 1) with the STR profile of the baby (cord blood). The detailed STR profile of the mother, her male baby and the cells isolated from the TCV are given in Supplementary Table S1. A similar STR profile analysis was done for the TCV cultures from a female baby and the results are shown in Supplementary Figure S4 and the corresponding Supplementary Table S2. (**d**) Immunofluorescence images showing a high CK18 and a low E-cadherin expression (scale bars, 50 μm). Image of the antibody negative control is shown in Supplementary Figure S5. (**e**) Flow cytometry analysis for MSC specific surface markers (first 6 columns) at passage 5 (mean ± standard deviation, n = 3). Histograms for each marker from a representative experiment are shown in Supplementary Figure S6. A relatively lower expression of MSC marker CD90 (84.8%) is noted here. (**f**) Tri-lineage differentiation of fetal MSC at passage 5 (scale bar, 100 μm). (**g**) GTG-banded chromosome analysis of male fetal MSC culture at passage 5 exhibiting a normal 46, XY karyotype. Corresponding metaphase spread is shown in Supplementary Figure S7.

TCV explants, plated for fetal MSC isolation, attained a spheroid like morphology within 3-5 days of culture and cells started migrating out from these spheroids (Supplementary online video S1 and S2). The migrated cells were harvested after 2-3 weeks of culture and expanded further. The cells (taken from passage 1 to 3) were analyzed for establishing their origin and characterized for their MSC properties (Fig. 3). The X and Y chromosome-specific FISH showed that all the cells expressed single X and Y specific signals (Fig. 3b) and the STR profile of isolated MSC, matched perfectly with that of the baby (Fig. 3c). This confirmed that these cells were exclusively of fetal (XY) origin. A lower expression of E-cadherin and a significant retention of CK-18 (Fig. 3d) indicated the possibility of an epithelial to mesenchymal transition (EMT) of the fetal MSC. This assumption is supported by the observation that, these cells initially (passage 1) had only 60% of CD90 marker expression which gradually increased to >90% during culture progress (data not shown). Immunophenotyping by flow cytometry at passage 5, showed the cells positive for all MSC markers, although the percentage of CD90 positive cells were slightly lower than that for the other markers (Fig. 3e). The cells were also capable of tri-lineage differentiation (Fig. 3f). We maintained the fetal MSC cultures up to passage 12 and karyotyping revealed a stable and normal karyotype (Fig. 3g). 7-AAD staining analysis by flow cytometry showed more than 90% cell viability at any tested time point.

**Figure 4:**
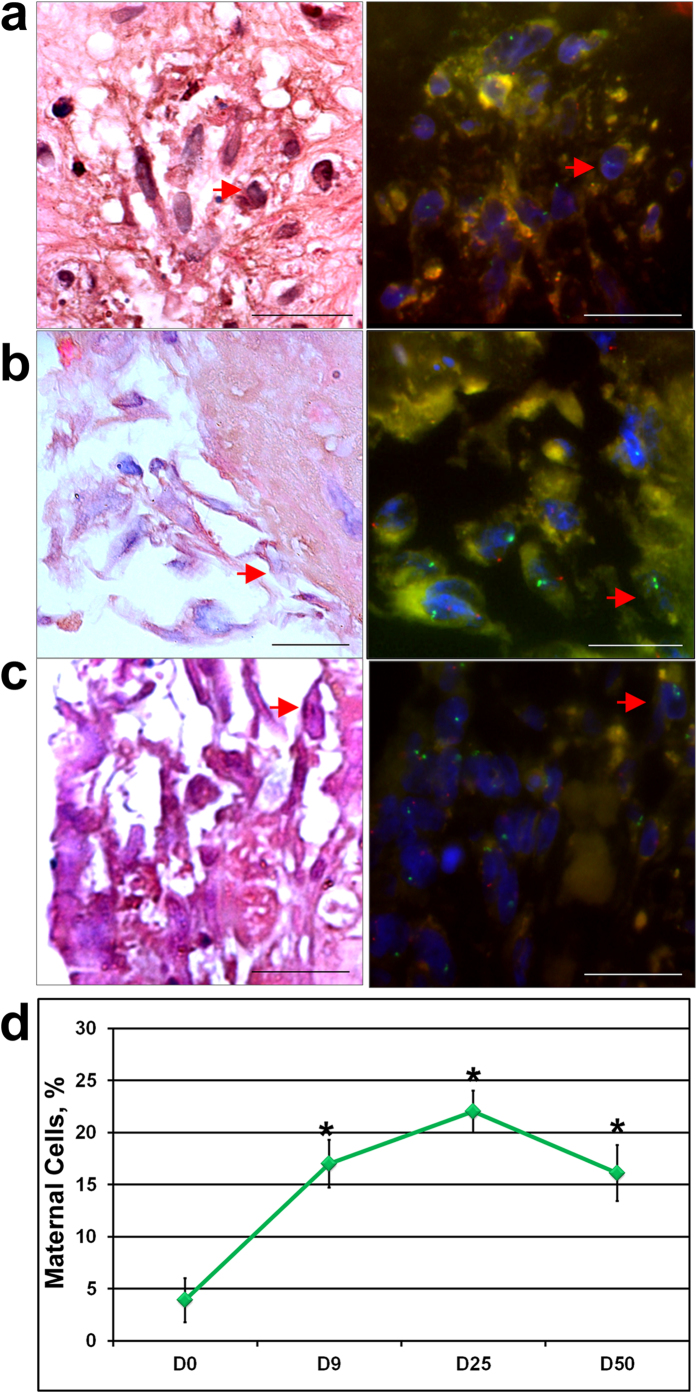
H&E staining and matched FISH analysis of TCV spheroid sections at different time points. (**a**) TCV spheroid sections after 9 days, (**b**) 25 days and (**c**) 50 days of culture. Red arrows shows cell of maternal origin. Majority of the cells migrating out of the spheroid edges were of fetal origin (**b**, **c**). (**d**) Graph showing the change in the percentage of maternal origin cells in the spheroids at different time points (in days, X axis). The data is represented as mean ± standard deviation (n = 3). Note the significant (p < 0.05) increase in the percentage of maternal cells from day 0 to day 25 of culture. Lower magnification images of the spheroid sections are shown in Supplementary Figure S8. Scale bars, 20 μm.

In order to confirm that migrating cells from the initial explants were exclusively of fetal origin, we enumerated the maternal and male fetal cells at different days of culture by FISH analysis in the residual spheroids of the original TCV explants (Fig. 4). The data clearly showed a significant increase (p < 0.05) in the percentage of maternal cells within the spheroids with the progress of culture period indicating that fetal origin cells preferentially migrated from the spheroid compared to the maternal cells (Fig. 4d). We noticed that by day 50, the cell migration from the spheroids had ceased.

### Maternal MSC characterization

**Figure 5:**
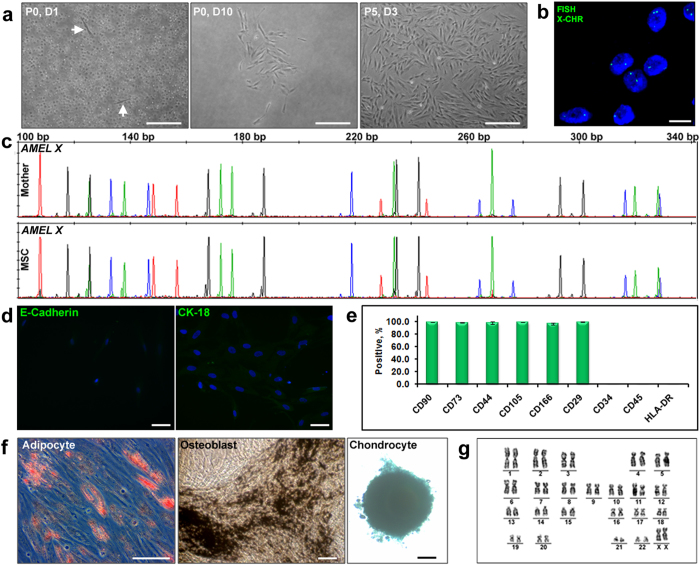
Cellular and molecular properties of MSC cultures established by Protocol 2. (**a**) Phase contrast images taken after 24 h of initial plating (P0, D1), 10 days (P0, D10) and on day 3 of passage 5 (P5, D3) are shown. (**b**) FISH images of adherent cell cultures at day 3 of passage 1 showing two X chromosomes-specific signals in all cells (a-b, scale bar, 10 μm). A similar FISH analysis was also done at passage 5 (Supplementary Figure S9). (**c**) Electropherograms showing the STR profiles of the mother and the adherent cell cultures (MSC) at passage 1. The STR profile of the MSC perfectly matched with the STR profile of the mother (maternal blood). (**d**) Immunofluorescence images of maternal cells at passage 1 showing low or no fluorescence for E-cadherin and CK18 markers (scale bars, 50 μm). Image of the antibody negative control is shown in Supplementary Figure S5. (**e**) Percentage of cells expressing MSC-specific surface markers (first 6 columns) as calculated by flow cytometry (mean ± standard deviation, n = 3). Histograms for each marker from a representative experiment are shown in Supplementary Figure S10. (**f**) Tri-lineage differentiation of MSC at passage 5 - adipocytes (Oil red O staining), osteoblasts (von Kossa staining) and chondrocytes (Alcian blue staining) (scale bars, 100 μm). (**g**) GTG-banded chromosome analysis of maternal MSC at passage 5 exhibiting a normal 46, XX karyotype. Corresponding metaphase spread for the karyotype is shown in Supplementary Figure S11.

The cells isolated by Protocol 2 were analyzed for establishing their MSC properties and maternal origin (Fig. 5). The majority of seeded cells remained non-adherent but a very small proportion (0.0001-0.0002%) could adhere to the tissue culture plate (Fig. 5a). The cells were harvested by trypsinization after 2-3 weeks of culture. We could propagate the adherent cells for several passages. The X chromosome-specific FISH (Fig. 5b) showed the presence of two X chromosome-specific signals and the STR profile of the isolated MSC (Fig. 5c) matched with the STR profile of the mother, confirming the maternal origin of these cells at passage 1. These cells at passage 1 lacked markers of differentiated epithelial cells such as; E-cadherin and cytokeratin-18 (CK-18) (Fig. 5d), indicting their indigenous mesenchymal nature. Immunophenotyping by flow cytometry (Fig. 5e) and tri-lineage differentiation to adipocytes, osteoblasts and chondrocytes (Fig.5f) confirmed that these cells exhibited all the properties of MSC defined by the International Society of Cellular Therapy^13^. We could maintain the MSC cultures up to passage 12 and the GTG banded chromosome analysis verified that they were karyotypically homogeneous and normal (Fig. 5g). 7-AAD exclusion of non-viable cells in flow cytometry analysis showed that more than 90% of the cells were viable at any tested time point.

Based upon these results it is very clear that adult and fetal cells prepared from the tips of TCV showed different physiological properties – while the fetal cells were more migratory, adult cells were more adherent to the substrates but both cell types yielded a similar set of mesenchymal stromal cell properties.

## Discussion

There are two related but independent aspects in this paper that are significant and require further investigation. First, we have shown that TCV-tips of normal human term placentas can be used for culturing fetal and maternal MSC from the same TCV. Propagation of stable and pure cultures of human fetal MSC has been a challenge and ours is the first successful method by which pure and stable fetal MSC cultures can be reproducibly derived from term human placenta. Although fetal MSC from other birth associated tissues have been reported^14^,^15^ but our method permits the derivation of fetal as well adult (maternal) MSC from the same tissue i.e. TCV. The mechanisms for the selection of fetal vs. adult MSC in our protocols need further understanding - in our experience adult (maternal) cells were more adhesive to plastic than fetal cells whereas fetal cells migrated faster than the maternal cells as evidenced by the significant increase in the proportion of maternal cells in the residual spheroids during the observation period (Fig. 4). Similar selective adhesion to plastic by other primary MSC obtained from other tissues has been documented^11^. Regarding the exclusive selection of fetal MSC by cell migration, we propose that fetal cells could migrate from the spheroids more effectively than maternal cells. The reduction of E-cadherin and the maintenance of CK-18 expression in fetal cells at passage 1 (Fig. 3) indicated that at this stage fetal MSC had undergone only a partial EMT, which reduced their inter-cellular binding and aided in their migratory properties. The complete acquisition of the mesenchymal phenotype by fetal cells was reflected in the expression of CD90 antigen, a marker very closely associated with mesenchymal cells. This indicated that the EMT of fetal cells was completed during the culture period. The molecular mechanism behind this selective migration of fetal cells is not clear, however a prior report on the growth of potentially fetal MSC from explants of chorionic villi^16^ supports our observation. The hypoxic environment of spheroids could have also played a role in enhancing the migration of cells^11^. The need for fetal cells, with multipotent / pluripotent differentiation potential, for regenerative medicine applications has been highlighted earlier^17^. The consistent and simple protocol for obtaining fetal MSC described in this paper would be able to fill in this demand. Our second significant finding is that, TCV also contain a small proportion (3-5%) of maternal cells, thereby indicating that the feto-maternal barrier in normal placentas is not totally impervious to maternal cell trafficking to the TCV. We could confirm the presence of maternal cells in TCV of all male placenta samples, but it could not be done in female placentas due to technical limitations of X chromosome FISH. Genotyping is an alternative method for demonstrating the presence of maternal cells within TCV of female babies. The possibility of maternal cell presence in TCV had been suggested earlier^6^ but no firm evidence was available. Therefore, the demonstration of maternal cells in normal TCV raises new questions about the control of the materno-fetal barrier in normal placentas The physiological significance of maternal cell trafficking in normal TCV is not clear, however some older reports have suggested that maternal inflammatory cells of immune origin could enter chorionic villi, in cases of villitis during pregnancy^18^.

## Conclusion

We have shown for the first time that maternal and fetal cells are co-localized in the tips of TCV; this observation emphasizes the necessity to reanalyze the nature of the feto-maternal barrier in term placenta. Although additional experiments are necessary to prove the full range of pluripotency and efficacy of pure fetal MSC from TCV for human therapeutic purposes, we have established a robust methodology to reproducibly obtain fetal MSCs from human tissue that is abundantly available and is ethically acceptable for human use. The importance of using fetal MSC in regenerative medicine has been emphasized in earlier reports^17^,^19^ and it has been proposed that fetal MSC would be more pluripotent and fast growing than adult MSC^20^, we therefore propose that fetal PMSCs shall be widely used for personalized stem cell therapies in the future. As we have not compared this fetal MSC from TCV with pure fetal MSCs from other available sources, further studies on these cells such as telomerase length, doubling time and expression of pluripotency related markers like Oct-3/4, Nanog, Rex-1, SSEA-3, SSEA-4, Tra-1-60 and Tra-1-81, need to be carried out. It would also be interesting to assess the ability of TCV derived fetal MSCs to differentiate into cells specific to the three germ layers i.e. ectoderm (epithelial and neuronal cells), endoderm (insulin producing β-cells and hepatocytes) and mesoderm (endothelial cells and cardiomyocytes).

## Methods

### Placenta

Approval of the Institutional Ethics Committee of Centre for Cellular and Molecular Biology (CCMB) was obtained for the sample collection, processing and other experimental procedures before initiating this experiment. All the experiments were carried out in accordance with the approved guidelines. Human placentas of uncomplicated, elective caesarean deliveries at term were collected after obtaining informed written consent from the mother. The placenta was processed in a sterile laminar hood by washing and carefully removing the inter-villus tissue to expose the white terminal chorionic villi (TCV) (Supplementary Fig.S1). The TCV was dissected out, minced and treated twice with 0.25% trypsin-EDTA (1 ml/g) in a magnetic stirrer at 37 °C for 10 min.

### Fetal MSC culture

The partially digested TCV obtained from the second trypsinization was further processed by Protocol 1 for isolating MSC of fetal origin. They were washed several times in DPBS and plated as explants in 0.1% gelatin coated tissue culture plates in MSC media (Knockout-DMEM medium supplemented with 10% fetal bovine serum (certified Australian, HyClone, Australia), 2 mM L-glutamine and 1% Pen Strep (10,000 units/ml Penicillin and 10,000 μg/ml Streptomycin)). The plates were incubated at 37 °C with 5% CO_2_ in a humidified incubator. The explants in culture attained a three dimensional spheroid like morphology within 3-5 days and will be described as spheroids henceforth. The cells were allowed to migrate out from the spheroids and complete media change was given every alternative day until the plates were ready for splitting (2-3 weeks). The cells were harvested using 0.25% trypsin-EDTA and propagated on 0.1% gelatin coated tissue culture plates.

All the cell culture media and reagents were purchased from GIBCO (Invitrogen, Life Technologies, USA) and the plastic wares were purchased from BD Biosciences (USA) unless specified otherwise.

### Maternal MSC culture

The cell suspension from the second trypsinization was further processed by Protocol 2 for isolating MSCs of maternal origin. The cells were suspended in MSC media and plated in tissue culture treated plates or flasks at a seeding density of 0.5 million cells per square cm area. The plates were incubated at 37 °C with 5% CO_2_ in a humidified incubator. The adherent cells were harvested using 0.25% trypsin-EDTA and propagated further. The cultures was observed at different time intervals in an inverted phase contrast microscope (Nikon Eclipse TS100, Nikon Instruments Inc., USA) and images were captured using Nikon Coolpix P6000 camera (Nikon, USA).

### Histocytochemistry

Dissected chorionic villi and spheroids collected at different time points (after 9, 25 and 50 days of culture) were formalin-fixed, embedded in paraffin blocks and serial sections were obtained using routine laboratory techniques. Consecutive sections were processed for hematoxylin-eosin (H&E) staining and fluorescence *in situ* hybridization (FISH) simultaneously. The stained slides were observed under microscope (Axioimager Z2, Carl Zeiss, Germany) and images were captured using Axiovision software (Carl Zeiss, Germany).

### Fluorescence *in situ* hybridization (FISH)

The paraffin sections on the glass slides were processed for FISH by the sodium thiocyanate (Sigma Aldrich, USA) according to the method mentioned elsewhere^21^. For performing FISH on cells cultured from the TCV, the cells were harvested using 0.25% trypsin-EDTA and processed according to the manufacturer’s instructions (Abott Molecular Inc., USA). The dehydrated slides were air dried and FISH was carried out using CEP® X Spectrum Orange™ / Y Spectrum Green™ DNA Probe Kit (Abott Molecular Inc., USA) according to the manufactures instructions. For each TCV sections, a minimum of 500 cells with proper signals from the captured images of 30 different fields were evaluated for the enumeration of cells of maternal (XX, female) and fetal (XY, male) origin. For spheroids at each tested time points, sections from 5 different spheroids, each containing a minimum of 50 cells from the captured images of 20 different fields were evaluated.

### Short Tandem Repeat (STR) profiling

Blood samples from the mother and her baby (cord blood) was collected after obtaining informed written consent of the mother. Genomic DNA from the blood and cultured cells were isolated by method mentioned elsewhere^22^. The extracted DNA was quantified using NanoDrop 1000 Spectrophotometer (NanoDrop Technologies, USA) and the purity was determined by measuring the 260/230 and 260/280 nm absorbance ratios. The probability of DNA degradation was evaluated by gel electrophoresis on 0.8% agarose gel.

STR profiles were generated from blood cells of the mother and her baby; and the cultured cells from the TCV using AmpFlSTR® Identifiler® PCR Amplification Kit (Applied Biosystems, USA), according to the manufactures instructions. Briefly, 1 ng of genomic DNA was used to amplify 16 STR loci in a multiplex reaction (Applied Biosystems® 2720 Thermal Cycler, Life Technologies, USA) and the PCR amplicons were analyzed in ABI Prism 3700 Genetic Analyzer (Applied Biosystems, Life Technologies, USA), using GeneScan software to obtain the allelic designation. The STR loci were detected by four different dye labels. D8S1179, D21S11, D7S820 and CSF1PO loci were detected by 6-FAM™ dye. VIC® dye detected D3S1358, TH01, D13S317, D16S539 and D2S1338 loci. NED™ dye detected D19S433, vWA, TPOX and D18S51 loci. PET® dye detected D5S818 and FGA loci. In addition to STR loci, this kit also consisted of Amelogenin locus (detected by PET® dye) to differentiate X and Y chromosomes.

### MSC characterization

MSC cultures were characterized by phase contrast microscopy, immunophenotyping and tri-lineage differentiation, X and Y chromosome–specific FISH, STR profile based genotyping and GTG banded karyotyping for establishing their mesenchymal properties and fetal / maternal origin. The cell cultures at different time intervals were observed under inverted phase contrast microscope (Nikon Eclipse-TS100, Nikon Instruments Inc., USA) and images were captured using Nikon Coolpix P6000 (Nikon, USA). Live imaging of the culture showing the migration of cells from the spheroids was captured using inverted microscope (Axiovert 200 M Live cell work station, Carl Zeiss, Germany) and the Axiovision software. The cells were fixed and stained for Anti-human primary antibodies of E-cadherin (raised in rabbit, Abcam, USA) and cytokeratin-18 (CK-18, raised in mouse, Sigma Aldrich, USA) according to the manufactures instructions and detected by FITC-conjugated secondary antibodies (Alexa Fluor®, Life Technologies, USA). The slides were observed under an epifluorescence microscope (Axioimager Z2, Carl Zeiss, Germany,) and images were captured using Axiovision software.

The cultured cells at different passages were tested for MSC markers by flow cytometry using the FACS antibodies like CD90, CD73, CD105, CD166, CD44, CD29, CD34, CD45 and HLA-DR for characterizing MSCs according to the manufactures instructions (BD Pharmingen, USA) and analyzed by flow cytometry in FACS Calibur (BD Biosciences, USA) using CellQuest™ software (BD Biosciences, USA) for 10,000 gated events. The cells were tested for their ability to differentiate into osteogenic lineages by method mentioned elsewhere^23^. Differentiation to adipogenic and chondrogenic lineages were carried out using StemPro® adipogenesis and chondrogenesis differentiation kit (GIBCO, Invitrogen, Life Technologies, USA) respectively according to the manufacturer’s instructions. Differentiation to osteoblasts, adipocytes and chondrocytes were confirmed by von Kossa, oil red O and Alcian blue staining respectively. The stained plates were observed under an inverted phase contrast microscope (Axiovert 200 M, Carl Zeiss, Germany) and the images were captured using Axiovision software.

### Chromosome analysis

The cytogenetic stability of the MSC cultures at passage 1 and 5 were verified by Giemsa-Trypsin G (GTG) banded karyotyping according to the method mentioned elsewhere^24^. The Giemsa stained metaphase spreads were imaged in a phase contrast microscope (Olympus BX51, Olympus, Japan); and analyzed using Cytovision software (Applied Imaging, UK). Ten metaphases were counted in each sample.

### Statistical analysis

18 placenta samples were collected and processed for establishing the differential isolation protocol for MSCs of fetal and maternal origin. For statistical significance, data from complete characterization and analysis of three placentas each from male babies and female babies were used here. Data of placentas from three male babies were used for calculating the male to female cell number ratios in FISH analysis of TCV sections. The statistical analysis was done by the two tailed, paired Student’s t test. In all the analysis a p-value less than 0.05 was considered significant and asterisks were given accordingly to indicate the level of significance as ^*^p < 0.05.

## Author Contributions

S.M. and G.P. conceived the idea. S.M., G.P., K.S.P., K.T. and K.L.R. designed the experiments and analyzed the data. K.S.P. provided the study material. S.M., K.L.R., M.K.K., A.G.R. and T.A.R. performed the experiments. S.M. and G.P. participated in discussing the results and in writing the manuscript. All authors reviewed the manuscript.

## Additional Information

**How to cite this article**: Mathews, S. *et al.* Propagation of pure fetal and maternal mesenchymal stromal cells from terminal chorionic villi of human term placenta. *Sci. Rep.* **5**, 10054; doi: 10.1038/srep10054 (2015).

## Supplementary Material

Supplementary Information

Supplementary Video 1

Supplementary Video 2
