# Supplementary material for: Propagation of pure fetal and maternal mesenchymal stromal cells from terminal chorionic villi of human term placenta
Source: Sci Rep. 2015 May 15;5:10054. doi: 10.1038/srep10054 (PMC4432313; doi:10.1038/srep10054)
Supplement: Supplementary Information [file srep10054-s1.pdf]

Supplementary information for

Propagation of pure fetal and maternal mesenchymal stromal cells from terminal chorionic villi of human term placenta

Smitha Mathews, K. Lakshmi Rao, K. Suma Prasad, M.K. Kanakavalli, A. Govardhana Reddy, T. Avinash Raj, Kumarasamy Thangaraj and Gopal Pande*

CSIR-Centre for Cellular and Molecular Biology, Uppal Road, Hyderabad 500007, India; Sridevi Nursing Home, [1]CSIR-Centre for Cellular and Molecular Biology, Uppal Road, Hyderabad 500007, India; Sridevi Nursing Home, Warasiguda, Hyderabad 500361, India and Prasad Hospital and Research Centre, Nacharam, Hyderabad 500076, India

*For contact, E-mail: gpande@ccmb.res.in

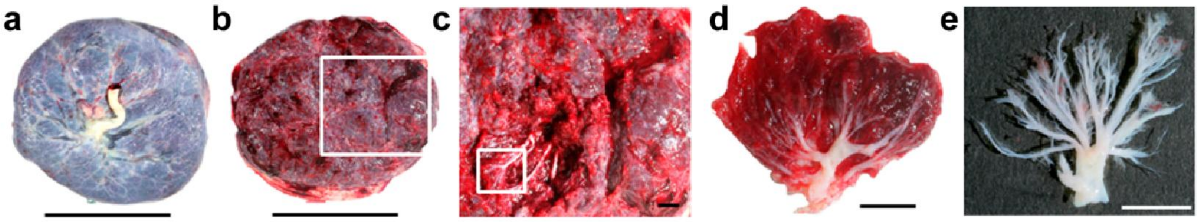

Supplementary Figure S1

Gross anatomy of a human placenta at full term: (a) Fetal side; (**b**) maternal side; **(c)** magnified view of maternal side of the boxed area in **b**, after removal of surface decidua; **(d)** dissected villus tree isolated from the boxed area of **c** before removal of inter-villus tissue and **(e)** villus tree as in **d** after removal and cleaning of inter-villus tissue. Scale bars, 10 cm (**a, b**) and 1 cm (**c-e**).

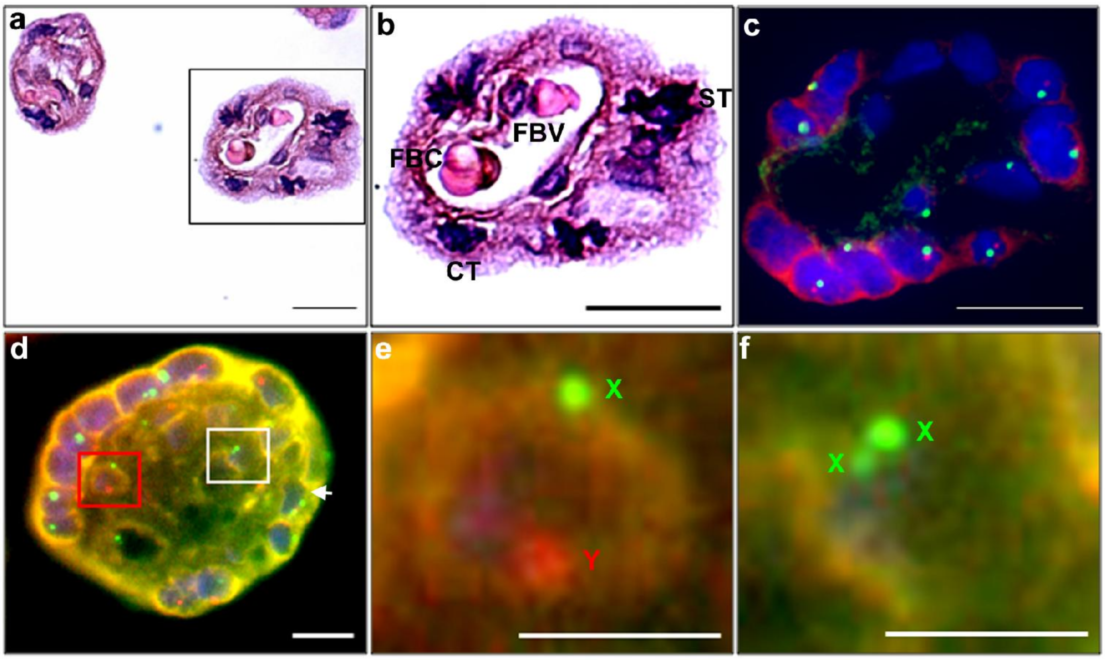

Supplementary Figure S2

Histological (H&E staining) and matched FISH analysis of maternal and fetal cells in the transverse section of the TCV: (**a**) H&E staining of a terminal villus (black square) and (**b**) the magnified image with the structural details: cytotrophoblast (CT), syncitiotrophoblast (ST), fetal blood vessel (FBV), and fetal blood cells (FBC). (**c**) The corresponding FISH based analysis of male fetal and maternal cells. All the cells in this villus showed fetal (XY, male) origin cells. (**d-f**) High-resolution FISH images demonstrating cells of both fetal and maternal origin. (**d**) FISH analysis showing the presence of male fetal (red square) and maternal (white square and arrow) cells. Majority of the cells were of fetal origin. (**f**) Higher magnification of **d** showed X and Y chromosome specific florescence for male fetal cells (red square) and two X chromosome specific florescence for maternal cell (white square). Scale bars, 50 µm (**a-c**) and 20 µm (**d-f**).

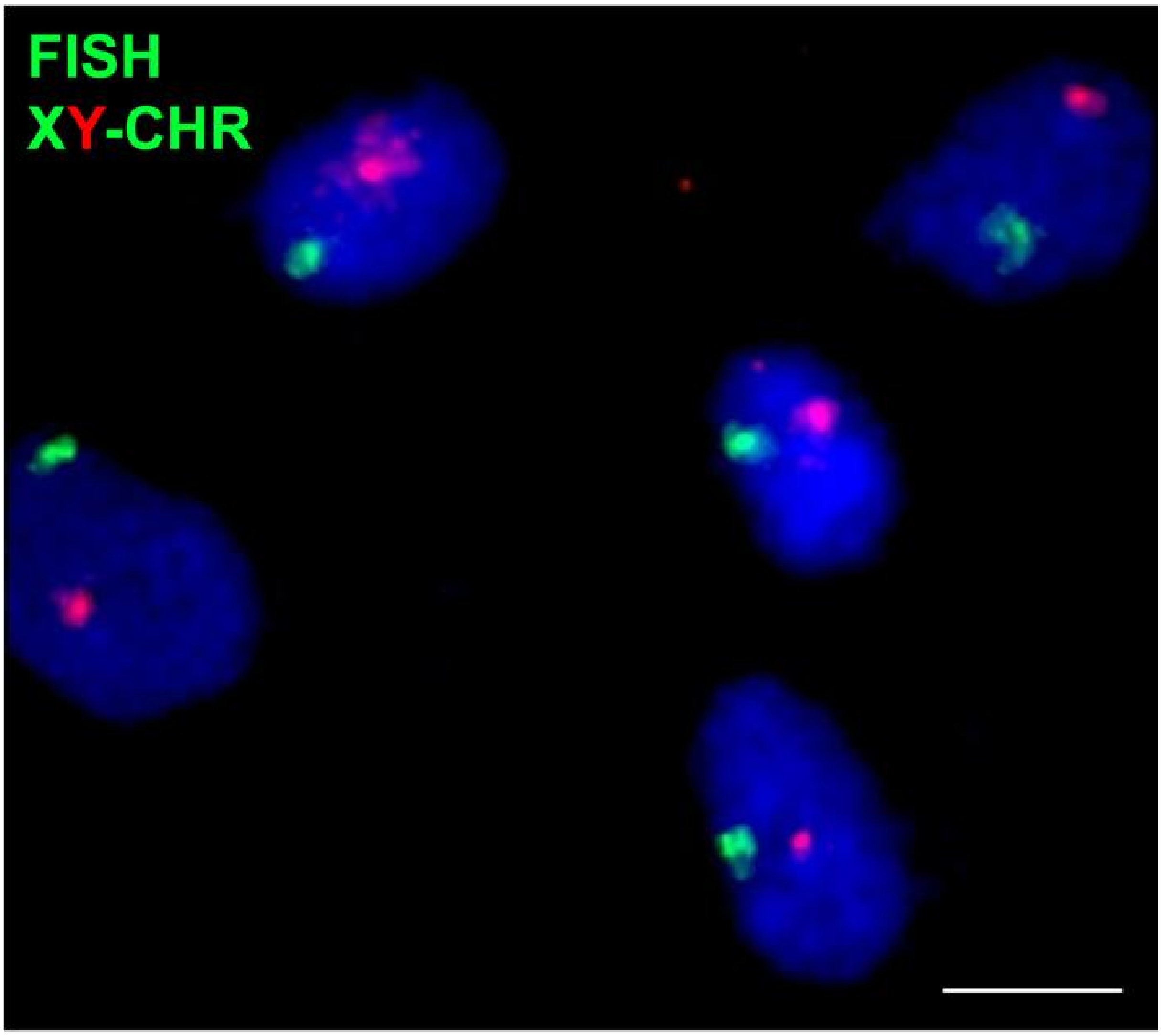

Supplementary Figure S3

FISH analysis of the cells isolated by Protocol 1 at later passage (P5): X and Y specific chromosomes show green and red fluorescence respectively. DAPI stained the nuclei blue. All the cells show X and Y-chromosomes (male cell), confirming their fetal origin. Scale bar, 10 µm.

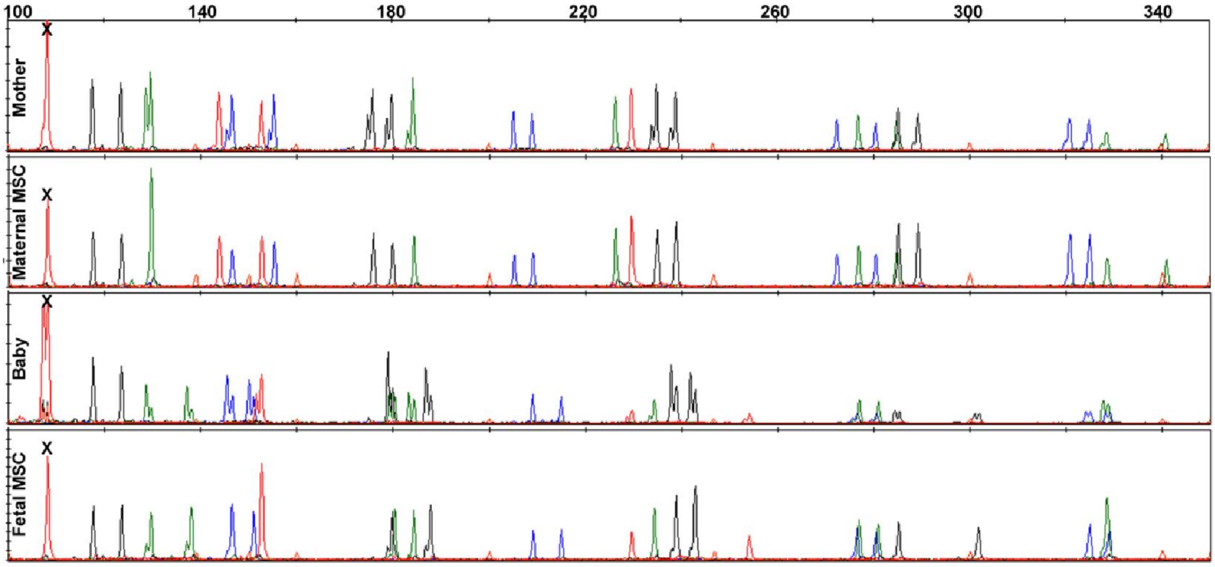

Supplementary Figure S4

Electropherograms showing the STR profiles of the mother, her female baby and the isolated cell cultures (MSC) at passage 1: The STR profile of the MSC isolated by protocol 1 (fetal MSC) matched perfectly with the STR profile of the baby (cord blood) indicating that the culture at passage 1 was entirely from fetal cells. The STR profile of the MSC isolated by protocol 2 (Maternal MSC) matched perfectly with the STR profile of the mother (blood sample) indicating that the culture at passage 1 was entirely from maternal cells. The Amelogenin locus showed a single peak (107 bp) corresponding to the X chromosome-specific (*AMEL* X) allele, for the mother, maternal MSC, baby and the fetal MSC indicating that the TCV was from a female baby. **Supplementary Table S2** shows the detailed STR profiles of the tested samples.

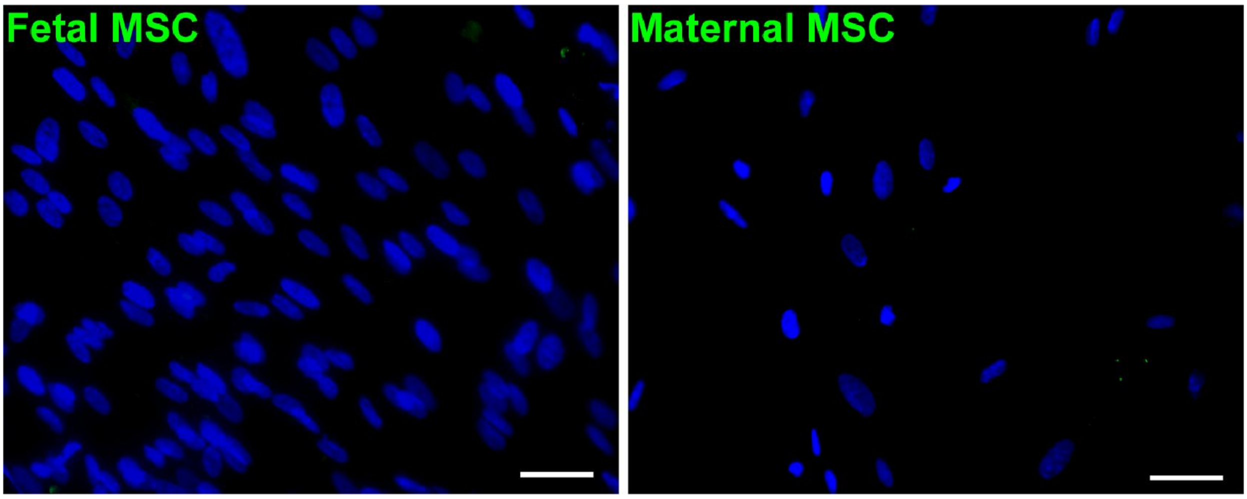

Supplementary Figure S5

Immunofluorescence images showing the antibody negative controls. Antibody negative controls for the mmunofluorescence staining for E-cadherin and CK18 expression in fetal and maternal MSC. Scale bar, 50 µm.

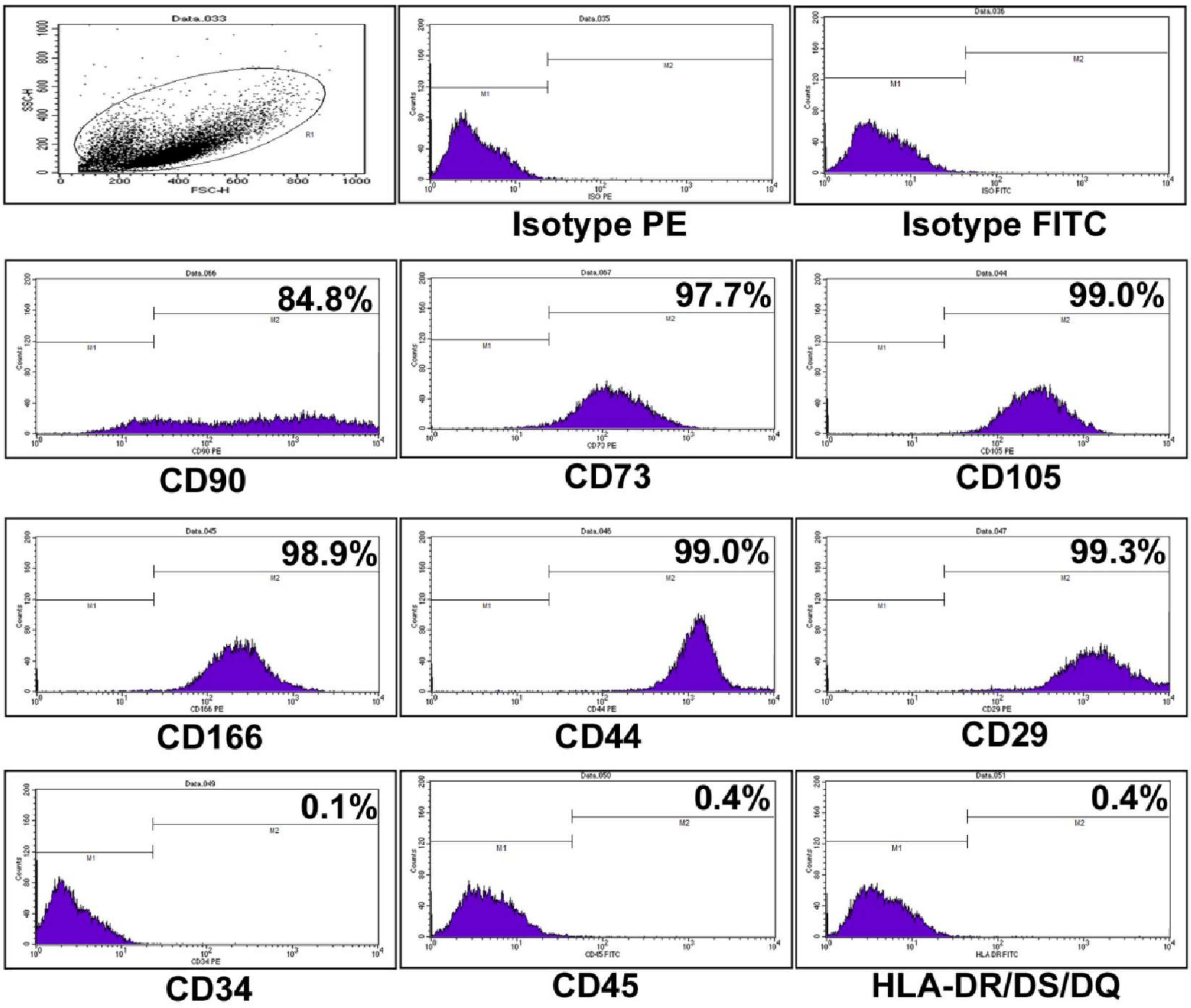

Supplementary Figure S6

Flow cytometry analysis for the MSC markers: The figure shows the representative data of the cells at P5, isolated by Protocol 1, from the placental TCV of a male baby. The dot plot shows the cell distribution and the histograms show the marker distribution. The marker names are mentioned below the histogram and the % of positive cells are mentioned in the right side corner of each histogram. The cells were high positive (≥95%) for mesenchymal stem cell markers like CD73, CD105, CD166, CD44, and CD29; and negative (≤2%) for hematopoietic markers like CD34 and CD45. The cells were also negative for HLA-DR. Reduced CD90 (84.4%) expression was noted in the cells isolated by Protocol 1.

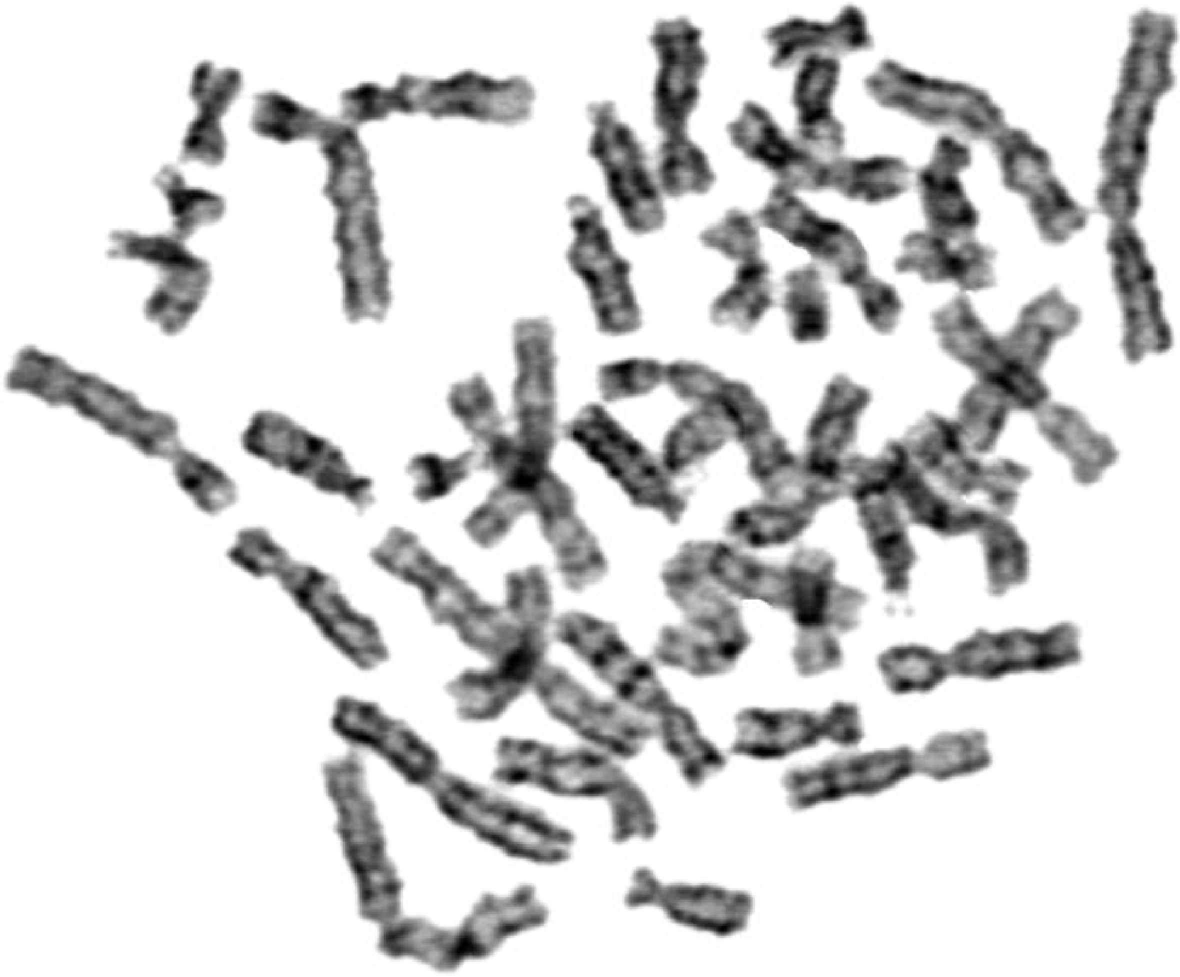

Supplementary Figure S7

GTG-banded karyotyping: The e figure shows the metaphase spread corresponding to **Fig. 3g** for the cells at P5, isolated from the TCV of a male baby by Protocol 1.

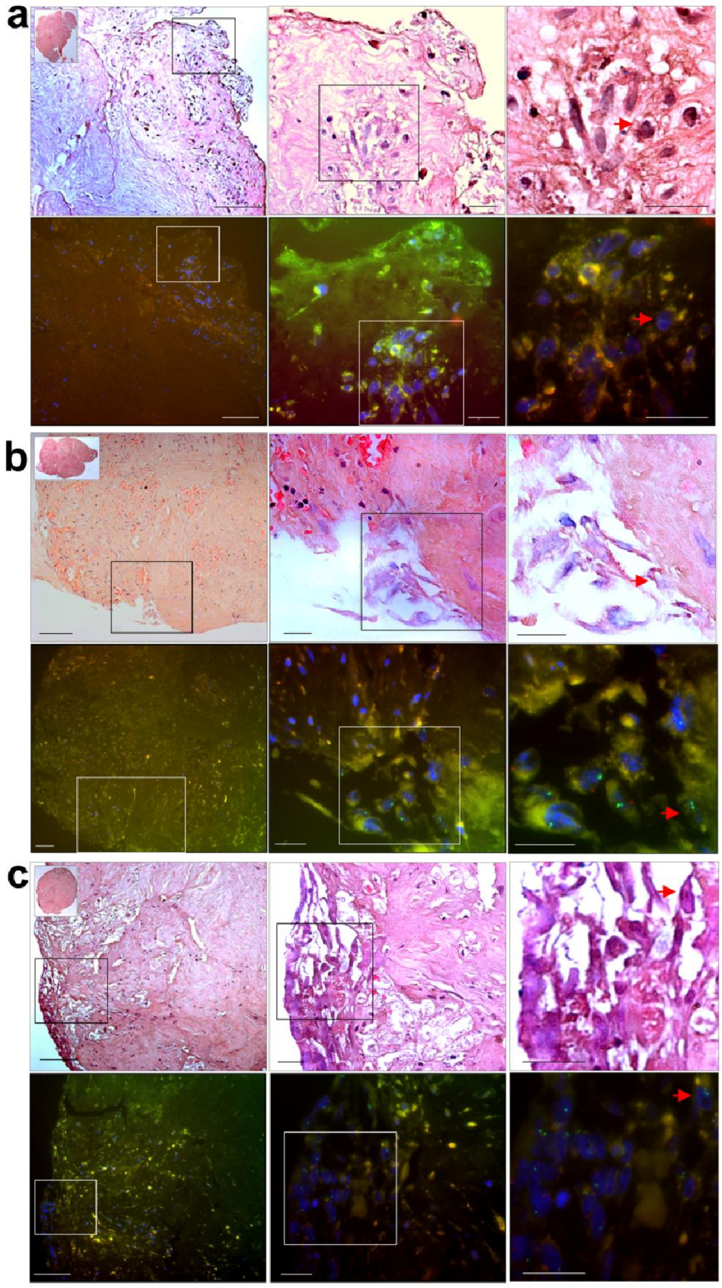

Supplementary Figure S8

H&E staining and matched FISH analysis of TCV spheroid sections at different time points: (**a**) TCV spheroid sections after 9 days, (**b**) 25 days and (**c**) 50 days of culture. Magnified images of the boxed area are shown in the following panel and red arrows are used to show cells of maternal origin. Inserts: Low magnification images showing the entire spheroid structure. Scale bar, 100 µm (first panel) and 20 µm (all other images).

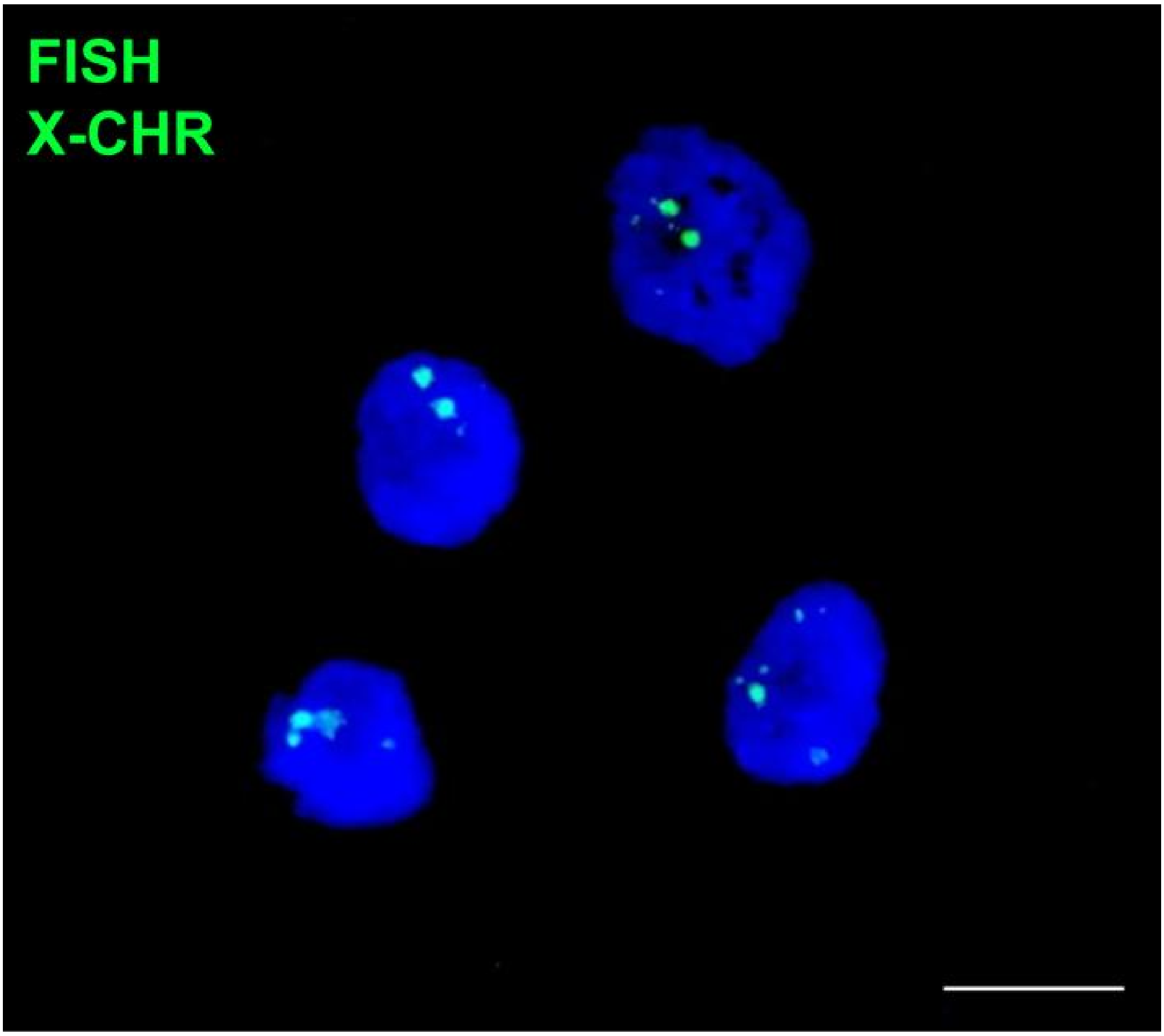

Supplementary Figure S9

FISH analysis of the cells isolated by Protocol 2 at later passage (P5): X specific chromosomes shows green fluorescence and DAPI stained the nuclei blue. All the cells shows two X chromosomes (female cell) confirming their maternal origin. Scale bar, 10 µm.

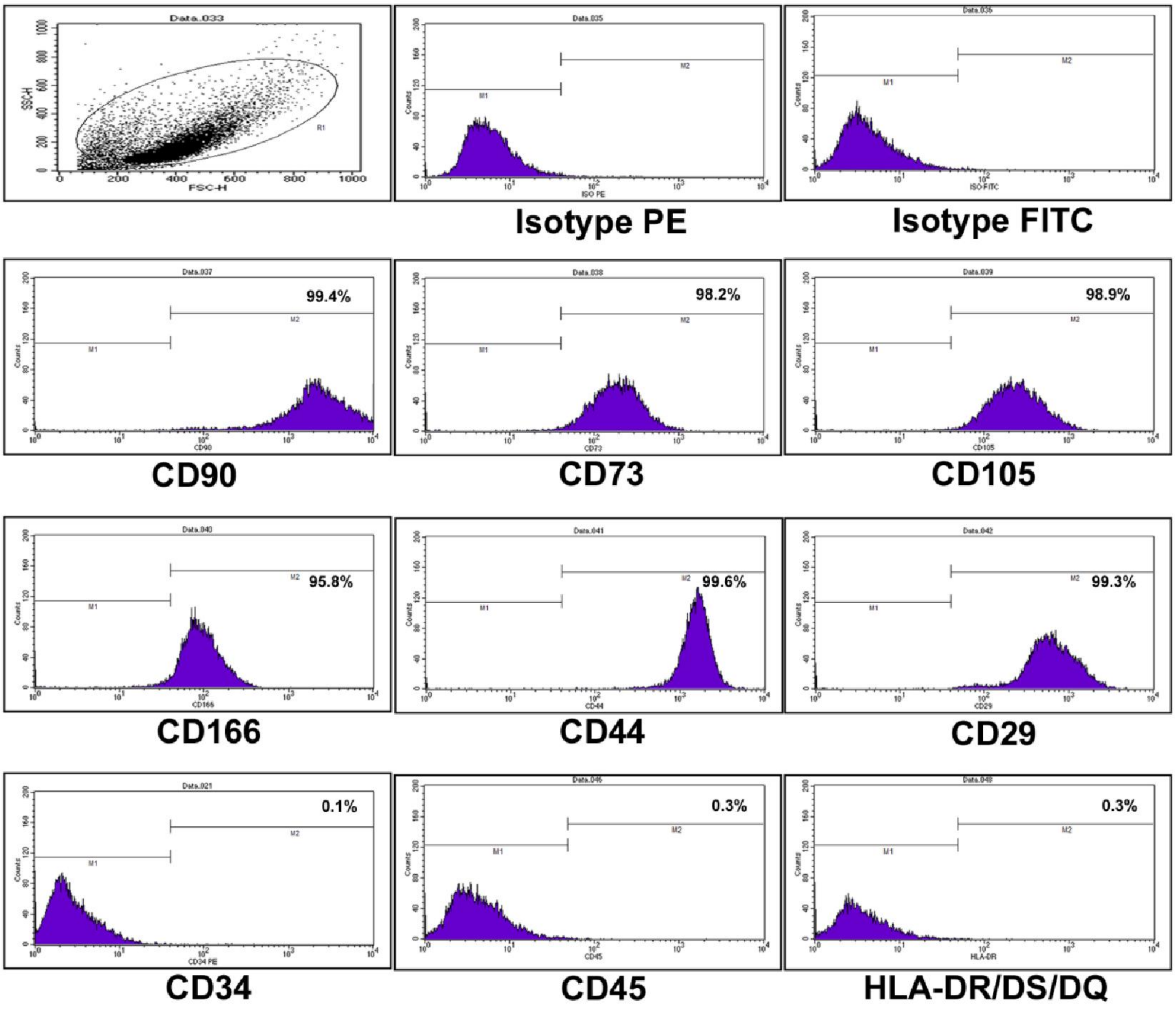

Supplementary Figure S10

Flow cytometry analysis for the MSC markers: The figure shows the representative data of the cells at P5, isolated by Protocol 2, from the placental chorionic villi of a male baby. The dot plot shows the cell distribution and the histograms show the marker distribution. The marker names are mentioned below the histogram and the percentage (%) of positive cells are mentioned in the right side corner of each histogram. The cells were high positive (≥95%) for mesenchymal stem cell markers like CD90, CD73, CD105, CD166, CD44, and CD29; and negative (≤2%) for hematopoietic markers like CD34 and CD45. The cells were also negative for HLA-DR.

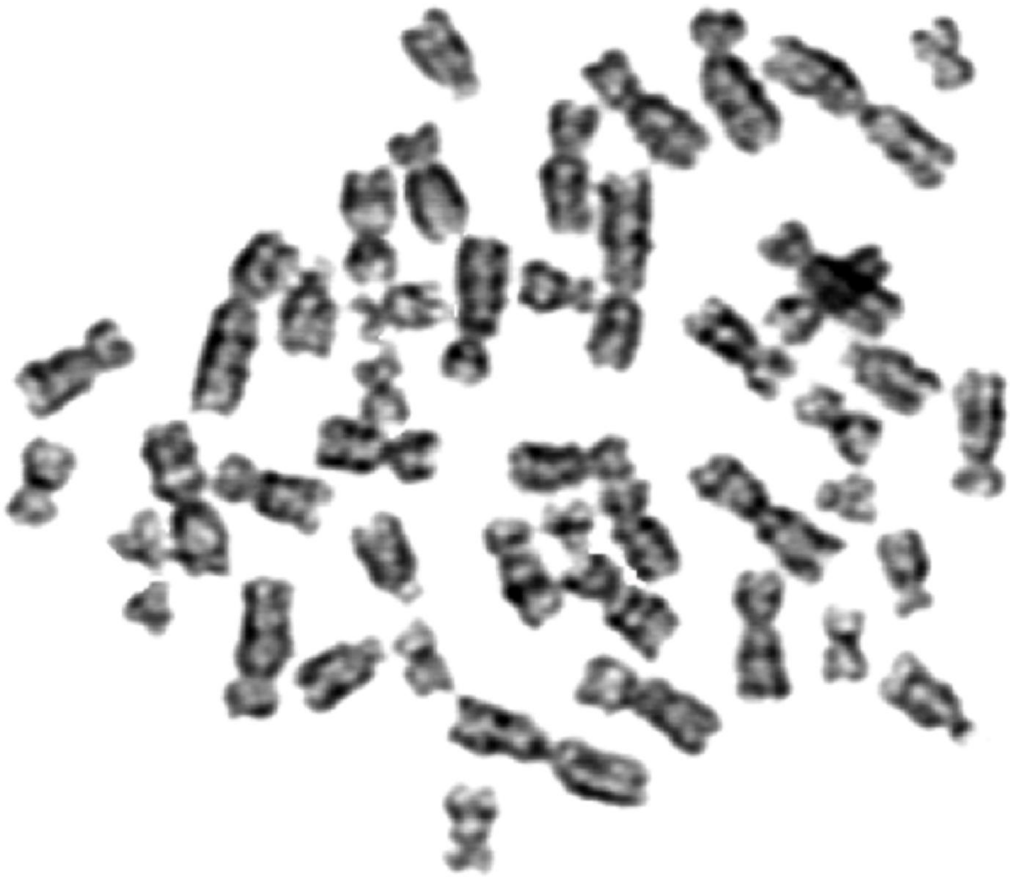

Supplementary Figure S11

GTG-banded karyotyping: The e figure shows the metaphase spread corresponding to **Fig. 5g** for the cells at P5, isolated from the TCV of a male baby by Protocol 2.

Supplementary Table S1

STR profiles of the mother, her male baby and the cells isolated from the TCV: The STR profile of the fetal MSC (Protocol 1) matched perfectly with the STR profile of the baby and the STR profile of the maternal MSC (Protocol 2) matched perfectly with the STR profile of the mother. Amelogenin confirms the presence of only X chromosome-specific allele (107 bp) for the maternal MSC, and both X (107 bp) and Y (113) chromosome-specific alleles for the fetal MSC.

Loci	Alleles	Mother	Maternal MSC	Baby	Fetal MSC
D8S1179	1<br>2	10<br>13	10<br>13	13<br>15	13<br>15
D21S11	1<br>2	32.2<br>32.2	32.2<br>32.2	31.2<br>32.2	31.2<br>32.2
D7S820	1<br>2	8<br>11	8<br>11	10<br>11	10<br>11
CSF1PO	1<br>2	9<br>12	9<br>12	9<br>12	9<br>12
D3S1358	1<br>2	15<br>18	15<br>18	17<br>18	17<br>18
THO1	1<br>2	6<br>7	6<br>7	6<br>9	6<br>9
D13S317	1<br>2	12<br>12	12<br>12	10<br>12	10<br>12
D16S539	1<br>2	9<br>9	9<br>9	9<br>11	9<br>11
D2S1338	1<br>2	18<br>20	18<br>20	18<br>18	18<br>18
D19S433	1<br>2	13<br>15	13<br>15	13<br>16	13<br>16
vWA	1<br>2	14<br>19	14<br>19	15<br>19	15<br>19
TPOX	1<br>2	9<br>11	9<br>11	8<br>11	8<br>11
D18S51	1<br>2	14<br>16	14<br>16	14<br>14	14<br>14
D5S818	1<br>2	10<br>12	10<br>12	10<br>10	10<br>10
FGA	1<br>2	20<br>24	20<br>24	19<br>20	19<br>20
Amelogenin	X/Y	X<br>X	X<br>X	X<br>Y	X<br>Y

Supplementary Table S2

STR profiles of the mother, her female baby and the cells isolated from the TCV: The STR profile of the fetal MSC (Protocol 1) matched perfectly with the STR profile of the baby and the STR profile of the maternal MSC (Protocol 2) matched perfectly with the STR profile of the mother. Amelogenin confirms the presence of only X chromosome-specific allele (107 bp) for both the maternal and fetal MSC as the TCV was from a female baby.

Loci	Alleles	Mother	Maternal MSC	Baby	Fetal MSC
D8S1179	1<br>2	13<br>15	13<br>15	13<br>14	13<br>14
D21S11	1<br>2	29<br>30	29<br>30	30<br>31.2	30<br>31.2
D7S820	1<br>2	10<br>12	10<br>12	11<br>12	11<br>12
CSF1PO	1<br>2	10<br>11	10<br>11	11<br>12	11<br>12
D3S1358	1<br>2	16<br>16	16<br>16	16<br>18	16<br>18
THO1	1<br>2	9<br>9	9<br>9	8<br>9	8<br>9
D13S317	1<br>2	10<br>10	10<br>10	12<br>12	12<br>12
D16S539	1<br>2	11<br>13	11<br>13	11<br>12	11<br>12
D2S1338	1<br>2	20<br>23	20<br>23	20<br>20	20<br>20
D19S433	1<br>2	13<br>14.2	13<br>14.2	13<br>14.2	13<br>14.2
vWA	1<br>2	16<br>17	16<br>17	17<br>19	17<br>19
TPOX	1<br>2	9<br>10	9<br>10	10<br>11	10<br>11
D18S51	1<br>2	12<br>13	12<br>13	12<br>16	12<br>16
D5S818	1<br>2	9<br>11	9<br>11	11<br>11	11<br>11
FGA	1<br>2	20<br>10	20<br>10	20<br>26	20<br>26
Amelogenin	X/Y	X<br>X	X<br>X	X<br>X	X<br>X
